# Case studies in Bayesian microbial risk assessments

**DOI:** 10.1186/1476-069X-8-S1-S19

**Published:** 2009-12-21

**Authors:** Marc C Kennedy, Helen E Clough, Joanne Turner

**Affiliations:** 1 Food and Environment Research Agency, Sand Hutton, York, YO41 1LZ, UK; 2 National Centre for Zoonosis Research, University of Liverpool, Leahurst, Neston, South Wirral, CH64 7TE, UK; 3 Department of Veterinary Clinical Science, University of Liverpool, Leahurst, Neston, South Wirral, CH64 7TE, UK

## Abstract

**Background:**

The quantification of uncertainty and variability is a key component of quantitative risk analysis. Recent advances in Bayesian statistics make it ideal for integrating multiple sources of information, of different types and quality, and providing a realistic estimate of the combined uncertainty in the final risk estimates.

**Methods:**

We present two case studies related to foodborne microbial risks. In the first, we combine models to describe the sequence of events resulting in illness from consumption of milk contaminated with VTEC O157. We used Monte Carlo simulation to propagate uncertainty in some of the inputs to computer models describing the farm and pasteurisation process. Resulting simulated contamination levels were then assigned to consumption events from a dietary survey. Finally we accounted for uncertainty in the dose-response relationship and uncertainty due to limited incidence data to derive uncertainty about yearly incidences of illness in young children. Options for altering the risk were considered by running the model with different hypothetical policy-driven exposure scenarios. In the second case study we illustrate an efficient Bayesian sensitivity analysis for identifying the most important parameters of a complex computer code that simulated VTEC O157 prevalence within a managed dairy herd. This was carried out in 2 stages, first to screen out the unimportant inputs, then to perform a more detailed analysis on the remaining inputs. The method works by building a Bayesian statistical approximation to the computer code using a number of known code input/output pairs (training runs).

**Results:**

We estimated that the expected total number of children aged 1.5-4.5 who become ill due to VTEC O157 in milk is 8.6 per year, with 95% uncertainty interval (0,11.5). The most extreme policy we considered was banning on-farm pasteurisation of milk, which reduced the estimate to 6.4 with 95% interval (0,11). In the second case study the effective number of inputs was reduced from 30 to 7 in the screening stage, and just 2 inputs were found to explain 82.8% of the output variance. A combined total of 500 runs of the computer code were used.

**Conclusion:**

These case studies illustrate the use of Bayesian statistics to perform detailed uncertainty and sensitivity analyses, integrating multiple information sources in a way that is both rigorous and efficient.

## Background

Quantitative Microbial Risk Assessment (QMRA) is a model-based tool for managing food chain risks and enhancing the science behind food safety regulations. QMRA models commonly describe complex farm-to-fork food production chains, involving uncertain parameters and processes. Interest concerns the impact that uncertainty in model inputs and structure can have on uncertainty in model outputs (estimates of "risk"). Bayesian statistics provides a unified approach for handling uncertainty and variability: by contrast with classical approaches it describes uncertainty probabilistically, permits strengthening of inference via incorporation of expert opinions, and synthesizes multiple types of uncertainty in a mathematical framework. Recent developments in Bayesian statistics, such as elicitation of expert opinions [1], Monte Carlo methods [2], and sensitivity analyses [3], have been applied in other fields and have great potential within QMRA.

## Methods

We present two new case studies as examples. The first quantifies the risk from Vero-cytotoxigenic *E. coli *(VTEC) O157 in pasteurised milk. The second illustrates an efficient Bayesian sensitivity analysis of a complex simulation model that estimates the prevalence of VTEC O157 within a managed dairy herd.

### Case study 1: dietary exposure to VTEC O157 from milk products

**Figure 1:**
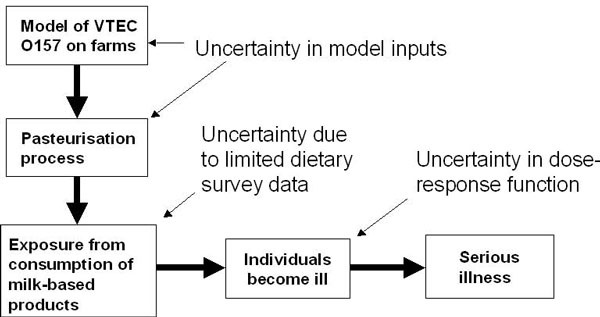
**Risk analysis model for Case Study 1**. Processes and uncertainties modelled in estimating the health risk from exposure to VTEC O157 in consumed milk products. Serious illnesses represent cases of Haemolytic uraemic syndrome.

As part of a collaboration with the RELU-RISK project [4], investigating stakeholder involvement in food chain risks, the processes leading to illness from VTEC O157 contaminated milk products were modelled (Figure 1). To estimate the rate of contamination in purchased milk products we used a process-based model of VTEC O157 prevalence in dairy herds and simulation models for pasteurisation failure in on- and off-farm pasteurisers. The contamination model, documented in [5], starts at the farm, with various inputs (herd prevalence, herd size, faeces amount per animal, proportion of animals lactating, prevalence in lactating animals, number of VTEC O157 colony forming units (cfu) per gramme in the faeces of infected animals, pasteurisation failure rate). The output of interest is the estimated number of cfu in a typical container of milk at point of sale. Variability between farms and animals is represented by stochastic simulation from probability distributions for herd size (negative binomial), faeces amount (gamma) and cfu/g of faeces (weibull). Parameters of these distributions were selected to approximately match available data.

To complete the exposure assessment data was taken on daily consumption of milk products of 1858 UK toddlers (aged 1.5-4.5) from the National Diet and Nutrition Survey (NDNS) [6] to be representative of the UK toddler population. The consumption and contamination models were linked by randomly assigning consumption events in the NDNS to either on- or off-farm contamination levels (*cfu/ml*), in proportion to the estimated market share of both types. As the market share for on-farm pasteurised milk is very small (around 0.16%), we separately generated results for the entire UK toddler population and for a 'high-risk' subpopulation of toddlers consuming milk only from on-farm pasteurisers. Sufficient milk contamination levels were generated to match the number of consumed items by running the code repeatedly, to represent the distribution of between-item variability. The total daily exposure *E*_*i *_was then calculated for each toddler together with the illness probability *p *= 1 - (1 + *E*_*i*_/*β*)^-*α *^[7]. Illness events were simulated using a Bernoulli distribution with probability *p*. Finally, approximate scaling factors were used to scale up from the NDNS-based incidences to the relevant populations.

Bayesian methods were used to quantify uncertainties due to limited information about some of the model inputs. For example, starting from uninformative beta(1,1) prior distributions for many of the rate and proportion inputs listed above, the posterior distributions were also beta distributed, after updating them using a sample of observed 'true' and 'false' outcomes. In each case, the amounts of data used in the updates determined the strength of information in the respective posterior distributions. The implied uncertainty in incidences was calculated via Monte Carlo simulation. The chain of models described above was repeated 1000 times with values for the uncertain inputs generated from their probability distributions. This included the posterior distribution of (*α*, *β*) derived in [7], so that a different relationship between dose and illness probability was used in each iteration.

#### Management options and how we modelled them

Key questions addressed by the study were whether on-farm pasteurisers present a particular problem, and if so what action should be recommended to reduce the risk. The modelling process was repeated for the following hypothetical scenarios, representing outcomes from possible management options.

1. Status Quo. The existing level of risk was estimated, based on current consumption levels and farming practice;

2. Ban on-farm pasteurisation, so that all consumed milk is assumed to come from off-farm pasteurisers;

3. Test more post-pasteurisation batches leaving a small dairy, effectively reducing the amount of contaminated milk by an amount depending on the accuracy of the test and the proportion of batches tested;

4. More training for on-farm pasteurisers. We assume that the ratio of human errors to mechanical errors (ordinarily assumed to be around 4:1) can be reduced through training down to 2:1 or 1:1, altering the off-farm pasteurisation failure rate accordingly. Risk managers could then decide how much reduction is desirable and practical.

### Case study 2: sensitivity analysis of a model for VTEC O157 transmission in a UK dairy herd

Our next case study used a deterministic SIR-type (Susceptible/Infective/Recovered) model to simulate the transmission of VTEC O157 within a typical UK dairy herd [8]. To capture the flow of infection around this structured population, the model included 4 interacting sub-groups (unweaned, weaned, dry and lactating), demographic processes and direct, indirect and pseudovertical (occurring in the first 24-48 h after birth) transmission. The result was a complex computer model containing 39 independent parameters. The aim was to identify key parameters related to prevalence of infection and suggest strategies for its reduction. The output of interest was prevalence of VTEC O157 in the weaned group, 150 days after the introduction of infection, as in [9].

As with many Bayesian methods for analysing computer codes [3,10] we assume the code output is an unknown function of its inputs (unknown in the Bayesian sense that before the code is run at a given input, the corresponding output is not available to the analyst). A Gaussian process prior distribution is assumed for this function [10], with a linear mean function that has unknown coefficients and a covariance between any pair of outputs that is a decreasing function of the distance between the corresponding inputs. This covariance represents a belief that the output is a smooth, continuous function of the inputs. After observing a series of selected code input-output (training) runs, the prior is updated to yield a posterior distribution for the function. This posterior is commonly referred to as the Bayesian *emulator *of the code. It includes the posterior mean, which is an approximation to the function, and a measure of residual uncertainty at any untried input. The emulation uncertainty is zero at the training inputs and increases smoothly at more distant points. Various uncertainty and sensitivity measures can be derived analytically from the emulator, if the input distributions are uniform or normal, without the need for additional code runs. These include variance based sensitivity measures that quantify the relative contribution to the total output variance due to variation in single inputs (main effects) and additional contributions to this variance due to variations in pairs of effects (interaction effects). A two-stage Bayesian sensitivity analysis [3] was carried out using the GEM-SA software [11] to investigate which of the uncertainties in the inputs contribute most to uncertainty in the output. The software is limited to 30 independent input parameters, so we first reduced the number of inputs to 30 by constraining a number of parameters to be common between groups (e.g. by making the recovery rate for the weaned group equal to the recovery rate for the unweaned group). In the first stage, 400 code runs were generated with inputs generated using the LP-Tau algorithm in GEM-SA. Since this was only a preliminary screening stage to identify those inputs that might possibly have a non-negligible influence on the output, uniform input distributions were assumed for all 30 inputs, with minimum and maximum values chosen to be conservative, based on the minimum and maximum values specified in [9]. These ranges represent a mixture of uncertainty and between-farm variability. A more detailed second stage sensitivity analysis was then carried out only considering the 'important' screened inputs (those contributing more than 0.5% to the total output variance). At this stage, more realistic distributions for the key inputs should be obtained, as the outputs are most sensitive to these and we would like a realistic assessment of the impact of genuine uncertainty in these inputs. Data analysis or methods for eliciting expert opinions [1] could be employed to obtain more detailed distributions, but this was not available in our study.

## Results

### Case study 1: dietary exposure to VTEC O157 from milk products

Each of the alternative management options considered is assumed to reduce the probability of a pasteurisation failure at an on-farm dairy and therefore reduce the probability of contamination in any single consumed item. Estimated yearly incidences are shown in Table 1. Uncertainty intervals for all scenarios include zero incidences. Although actual incidences in a given year may indeed be zero, the true expected annual incidence is greater than zero. Zeros arise from rounding errors and from the limited number of iterations in the simulation.

**Table 1:** Risk estimates for Case Study 1

**Toddler group & scenario**	**Estimated incidence (per year)**	**95% credible intervals**	
Current UK toddlers	8.6	0	11.5
Ban on-farm	6.4	0	11
**Consumers of on-farm milk products only**			
Current UK toddlers	1.7	0	7.3
Testing (50% reduction)	0.8	0	4.7
Training (40% reduction)	1	0	5.3
Training (60% reduction)	0.6	0	1.9

Estimated yearly illnesses for the UK toddler population and for the sub-population of on-farm consuming toddlers. Credible intervals quantify uncertainty in the total yearly incidences. The % reduction levels refer to the amounts assumed for the reduction in pasteurisation failure levels for a given policy scenario. For example, a 50% reduction is achieved if all batches are tested and the test has a 50% success rate in identifying contaminated batches.

### Case study 2: sensitivity analysis of a model for VTEC O157 transmission in a UK dairy herd

Of the initial 30 inputs, only 7 were found to contribute more than 0.5% to the total output variance. This screening of important input parameters meant that a more detailed investigation could be carried out at the second stage. A smaller input design was created, using 100 code runs but now allowing the 'common' inputs to vary independently. For example, the restriction from Stage 1 was relaxed so that the weaned and unweaned group were assumed to have distinct recovery rates. The new emulator was built using only the 8 sensitive inputs with the remaining inputs fixed at their default values. The uniform distributions were unchanged from the first stage. The effects of just 2 inputs, including the interaction effect based on this pair, explained 82.8% of the output variance. The inputs are recovery rate (i.e. the inverse of the duration of infection), which contributed 54%, and concentration of VTEC O157 in faeces, which contributed 22.6%. Both inputs have an impact on the level of indirect transmission (i.e. transmission via the environment). The joint effect contribution was 6.2%. The implication is that efforts to reduce prevalence in the weaned group should focus on changing the values of these parameters. Measures such as vaccination [12] and dietary management [13,14] can reduce the level and duration of shedding of VTEC O157 in cattle. Our results suggest that these should be more successful than alternative control measures (e.g. improving hygiene). A more conventional sensitivity analysis of this model, using partial rank correlation coefficients, is reported in [9], and was based on 1000 runs of the model. Our 2-stage Bayesian approach achieved similar results based on a total of 500 runs.

## Conclusion

The case studies presented here have demonstrated some of the potential uses for Bayesian approaches in QMRA to integrate information sources in an efficient, rigorous way. In Case Study 1, multiple sources of uncertainty are propagated through a farm-to-fork model to produce uncertainty in the exposures for the population. This is coupled with uncertainty derived from a Bayesian dose-response model to produce uncertainty intervals for health outcomes that are relevant to policy-makers and other stakeholders (Table 1). Case Study 2 showed how Bayesian sensitivity analysis could be used to understand the influence of uncertain inputs, in a way that is far more efficient than conventional Monte Carlo simulation.

## Note

The peer review of this article can be found in Additional file 1.

## Competing interests

The authors declare that they have no competing interests.

## Authors' contributions

MK carried out statistical analyses for both case studies, contributed to the design of the risk analysis model and drafted the manuscript. HC contributed to the design of the risk analysis model, implemented the simulation model and organised the stakeholder workshops for Case Study 1. JT contributed to the design and implementation of Case Study 2. All authors read and approved the final manuscript.

## Supplementary Material

Additonal file 1 Peer review. Click here for file
