# Supplementary material for: Case studies in Bayesian microbial risk assessments
Source: Environ Health. 2009 Dec 21;8(Suppl 1):S19. doi: 10.1186/1476-069X-8-S1-S19 (PMC2796497; doi:10.1186/1476-069X-8-S1-S19)
Supplement: Additonal file 1 — Peer review. [file 1476-069X-8-S1-S19-S1.doc]

| Article ref no. | **A1471-2105X SZ-S19** |
| --- | --- |
| Title | **Case Studies in Bayesian microbial risk assessments** |
| Author(s) | Marc Kennedy, Helen Clough and Joanne Turner |

**Ref#1**

**General comments:**

The English in the paper is mostly very good.

**Major compulsory revisions:**

My major problem with this paper is that it doesn’t explain what they’ve done. This seems fairly fundamental to me. As a reader of the paper, I should be able to reproduce what has gone on or, at the very least, be able to critically comment on what the authors have done. I don’t like the ‘black box’ statistical approach (we found some software, bunged some stuff in it and here is what came out the other end) that seems to be adopted here. At the very least, the readers need to know how the black box works so that they can try the same thing in the same or an alternative black box.

I will concentrate my more detailed comments on the first study. The Methods section seems to be where they can improve most. Presumably they have a model that relates the probability of a toddler being ill from VTEC 0157 to a number of ‘inputs’. I think we should be told what those inputs are. We could also do with some details of how the software works at this stage. My guess is that each of the inputs is assigned a probability distribution (we are told that these are taken as Uniform) and that the uncertainty comes from repeat sampling from these distributions. The form of the probability distribution is probably important – so we need to know why they chose Uniform distributions and not some other sort. This is where the expert judgement should come in, perhaps.

Generally, one way in which they could test the sensitivity of their results is to see how sensitive they are to the input distributions used. Of course, if they are sure that Uniform distributions are the right ones to use then they should say why.

Page 3, line 5. They introduce the concept of training runs. They should really explain what they mean by this.

I want to know what the model is because that is what the whole paper rests on. We just have to take their word that they have done the right thing. They do mention Quantitative Microbial Risk Assessment – but this doesn’t help the reader much. I want to know what their food-to-fork model is. If the whole thing is too complicated to explain here then i want to be pointed to somewhere where it has been explained. But i want some explanation here at the very least.

Page 3, line –3. I would delete use of the word ‘spectacular’. Statisticians are conservative creatures (with a small ‘c’) and most would wince at this word (I certainly did). I don’t even think it’s true.

I note that the uncertainty intervals all seemed to contain 0. I wonder why this is? Deserves a comment from the authors I would have thought.

Page 5. The scenarios seem sensible (though I am a statistician rather than a milk expert).

Page 7. They mention ‘code runs’. What are these? (they don’t define ‘common’ either as far as i can see). They also mention ‘the interaction effect based on this pair’. Without knowing what the model is, i have no idea what ‘interaction’ means in this context.

Page 8. They say that Case Study 2 shows how Bayesian sensitivity analysis is more efficient than Monte Carlo simulation. Without knowing what the model is, this claim seems meaningless. From what I can read in the paper, there is no evidence that their model is even Bayesian. The last sentence seems a little odd. They just list 3 topics that they say are relevant. I’d like some more explanation. What does extreme value theory have to do with all this, for example?

**Ref#2**

**General comments;**

This is an interesting and potentially very useful paper. However, the lack of detail in explaining the methods and in the risk model used in this study needs to be addressed by the authors before the paper can be considered as suitable for publication.

**Responses to Referees’ Comments**

**Ref#1**

**General comments:**

The English in the paper is mostly very good.

**Major compulsory revisions:**

My major problem with this paper is that it doesn’t explain what they’ve done. This seems fairly fundamental to me. As a reader of the paper, I should be able to reproduce what has gone on or, at the very least, be able to critically comment on what the authors have done. I don’t like the ‘black box’ statistical approach (we found some software, bunged some stuff in it and here is what came out the other end) that seems to be adopted here. At the very least, the readers need to know how the black box works so that they can try the same thing in the same or an alternative black box.

# Response to this has been to add more details of the farm-to-retail model of contamination in milk products. This includes a list of the main inputs and the distributions used for these. More details of the simulation implementation has also been included.

# I have also added a section on Bayesian emulation and sensitivity analysis in the methods section of Case Study 2. GEM-SA is an implementation of the Bayesian sensitivity analysis (SA) and is also used in the papers referred to (reference to O’Hagan, 2006, has been added, as this is appropriate for a reader unfamiliar with the concepts used in this use of Bayesian analysis for computer models)

I will concentrate my more detailed comments on the first study. The Methods section seems to be where they can improve most. Presumably they have a model that relates the probability of a toddler being ill from VTEC 0157 to a number of ‘inputs’. I think we should be told what those inputs are. **The main model inputs are now listed in the text. A reference to a more detailed description is also included [5]. This reference has now been published, so details have been updated. Dose (daily cfu intake) is computed by assigning a contamination level from either the on- or off- farm models. The probability of being ill, given this daily dose, is taken from the dose-response analysis described in [7]. The authors provided us with simulations directly from their analysis, which we then used in our study. Details of these steps have been added/improved in the methods section for Case study 1.**

We could also do with some details of how the software works at this stage. **Added details on Bayesian SA in methods section.** My guess is that each of the inputs is assigned a probability distribution (we are told that these are taken as Uniform **– Uniform distributions are used in case 2, but not case 1. Details of the distributions used in case 1 are now included in the extra detail added** and that the uncertainty comes from repeat sampling from these distributions. **Yes,** **Monte-Carlo modeling is mentioned in the abstract, and is the standard approach to propagating uncertainty through computer models.** We have **added more text towards the end of the methods text for Case 1**. The form of the probability distribution is probably important – so we need to know why they chose Uniform distributions and not some other sort. This is where the expert judgement should come in, perhaps. In Case 2, where uniform input distributions are used in the first stage. **I have added a reference to expert opinion for stage 2, as it may be used for more refined input distributions.**

Generally, one way in which they could test the sensitivity of their results is to see how sensitive they are to the input distributions used. Of course, if they are sure that Uniform distributions are the right ones to use then they should say why. **I have explained that the initial screening stage does not require very accurate distributions, and for the unimportant inputs, the distribution form has very little impact anyway. However in the final stage (e.g. follow on from stage 2) care should be taken as the distributions for the important inputs is expected to have most impact, and expert opinions are potentially useful here to obtain a suitable distribution.**

Page 3, line 5. They introduce the concept of training runs. They should really explain what they mean by this. **Expanded text.**

I want to know what the model is because that is what the whole paper rests on. We just have to take their word that they have done the right thing. They do mention Quantitative Microbial Risk Assessment – but this doesn’t help the reader much. I want to know what their food-to-fork model is. If the whole thing is too complicated to explain here then i want to be pointed to somewhere where it has been explained. But i want some explanation here at the very least.

# Details on the milk pasteurization model (see above) should answer this

Page 3, line –3. I would delete use of the word ‘spectacular’. Statisticians are conservative creatures (with a small ‘c’) and most would wince at this word (I certainly did). I don’t even think it’s true. **Deleted.**

I note that the uncertainty intervals all seemed to contain 0. I wonder why this is? Deserves a comment from the authors I would have thought. **Added comment in results section for Case 1.**

Page 5. The scenarios seem sensible (though I am a statistician rather than a milk expert).

**Thank you.**

Page 7. They mention ‘code runs’. What are these? **Added text to clarify, these are runs of the computer code that we are trying to approximate (also referred to as training runs). The code runs are the data used in the Bayesian updating of the Gaussian process approximation.** (they don’t define ‘common’ either as far as i can see) **The common inputs are described shortly after the introduction to GEM-SA, when explaining how the number of inputs was initially reduced**. They also mention ‘the interaction effect based on this pair’. Without knowing what the model is, i have no idea what ‘interaction’ means in this context. **More explanation related to Bayesian SA added.**

Page 8. They say that Case Study 2 shows how Bayesian sensitivity analysis is more efficient than Monte Carlo simulation. Without knowing what the model is, this claim seems meaningless. From what I can read in the paper, there is no evidence that their model is even Bayesian. **The new introduction to the methods section for Case 1 adds details about the Bayesian method.** The last sentence seems a little odd. They just list 3 topics that they say are relevant. I’d like some more explanation. What does extreme value theory have to do with all this, for example? **Deleted the last sentence. I agree it doesn’t add anything so this frees up some space. Elicitation is relevant to the case studies, so has been referred to earlier in the text where appropriate.**

**Ref#2**

**General comments;**

This is an interesting and potentially very useful paper. However, the lack of detail in explaining the methods and in the risk model used in this study needs to be addressed by the authors before the paper can be considered as suitable for publication.

**The additional details have been added on the risk model (Helen) and methods as detailed above.**

# General response from the authors to referees comments

Essential details on the methods used has been added to the Methods section of the paper, especially in relation to the process model for the farm to retail risk model used in Case Study 1 and for the Bayesian statistical method applied in Case Study 2. We have tried to balance the amount of additional detail with the need to save space. Further details (and the actual R code used in Case study 1) are available from the corresponding author. The statistical software GEM-SA used in Case Study 2 was written by the first author, and its workings are described in detail in the references [3, 10, 11]. Additional details have been added to introduce the essential features of the method, allowing for an assessment of its suitability for this application without reference to the other papers. The Bayesian nature of the method has also been made clearer. Monte Carlo sampling, in which a probability distribution is assigned or derived for the model inputs and realizations from these input distributions are used to run the model, is used to propagate uncertainty in Case Study 1. For Case Study 2 this is not necessary, as explained in the new Methods section. Here the uncertainty and sensitivity measures are computed analytically within the Bayesian analysis. This analytical calculation is only available from the GEM-SA software when input distributions are uniform or normal, and this restriction is stated in the Methods section.

The introduction to Case Study 2 in Methods has been rewritten to give more technical details of our method. An additional comment has been added to explain why Case Study 1 uncertainty intervals for yearly incidences all include 0. In the Results section for Case Study 2 we have also added an example of how the common inputs were separated at the 2^nd^ stage analysis.

Uniform distributions were used to represent parameter uncertainty in the herd transmission model of Case study 2. We have explained that the initial screening stage does not require very accurate distributions and it is more important that the ranges are chosen to be conservative. For the unimportant inputs, the distribution form has very little impact anyway. However in the final stage care should be taken as the distributions for the important inputs is expected to have most impact, and expert opinions are potentially useful here to obtain a suitable distribution. Unfortunately these were not available within our study.

Other specific points made by the referees about the clarity of the paper have been addressed. Some unnecessary detail has been removed or moved to a more appropriate part of the manuscript. An example is the final sentence, including the reference to expert elicitation methods, which is actually most relevant to the second stage analysis of Case Study 2.
